# Gender in mental health: toward an LGBTQ+ inclusive and affirming psychiatry and mental healthcare in the Philippines

**DOI:** 10.3389/fpsyt.2023.1189231

**Published:** 2023-06-22

**Authors:** Rowalt Alibudbud

**Affiliations:** Department of Sociology and Behavioral Sciences, De La Salle University, Manila, Philippines

**Keywords:** sexual and gender minorities, LGBT persons, gender affirmation, Mental Health Act, gender equality, Philippines, SOGIE Equality Bill, mental health

## 1. Introduction

The Philippines enshrined in its constitution the commitment to uphold the rights of all people regardless of gender (1–3). Toward this commitment, it has been considered the most gender-equal country in Asia in the Gender Gap index (4). However, discrimination against lesbians, gay men, bisexuals, transgenders, queers, and other individuals with diverse genders and sexual identities (LGBTQ+) remained persistent (1–3). In addition, negative attitudes toward gay men and lesbians have been reported by about a quarter of the Filipino population over the years (5–7). Moreover, a study involving Filipino students found that non-gender-variant participants reported more genderism and transphobia than their gender-variant peers (8). Furthermore, another study found that these negative societal attitudes may translate to high self-stigma among LGBTQ+ individuals (9).

The continued negative attitudes against LGBTQ+ Filipinos and their self-stigma can lead to higher rates of mental disorders (1, 9–21). Therefore, there is a need for mental healthcare that is responsive to the needs of LGBTQ+ Filipinos. However, these negative attitudes toward LGBTQ+ Filipinos have also been reported among mental health professionals. For instance, a Filipino psychologist on a national television show advised parents with LGBTQ+ children to consider conversion therapy to achieve a “happy family life” despite evidence of its deeply damaging consequences (10). These negative attitudes are further complicated by the limited number of trained counselors who can address their depression, suicidal tendencies, self-acceptance, anger, and family relationship issues, as highlighted by a UNDP and USAID report (2). Thus, in accordance with the Philippine Mental Health Act's guaranteed rights and protection from sexual orientation, gender identity, and expression (SOGIE)-based discrimination of service users (22), this opinion paper discusses several steps that can be undertaken to achieve inclusive and affirming mental healthcare for LGBTQ+ people in the country.

## 2. Negative attitudes and discrimination against LGBTQ+ Filipinos

Negative attitudes and discrimination against LGBTQ+ Filipinos may stem from Philippine heteronormative norms that promote a gender binary perspective (1, 2, 5–7). In this perspective, only men and women are acknowledged, disregarding the spectrum and diversity of SOGIE (1, 2, 5–7). These norms are rooted in colonial, religious, and cultural factors. Furthermore, they are reflected in Philippine societal positions, laws, and attitudes toward LGBTQ+ individuals (1–3, 5–8). For instance, the proposed SOGIE Equality Bill, which seeks to penalize SOGIE-based discrimination, institute redress mechanisms for discrimination, and establish programs that promote non-discrimination and diversity, has languished in the Philippine Senate for about 20 years (1, 2, 23). The most common argument against this bill is religious immorality, including some politicians who consider LGBTQ+ Filipinos 'worse than animals' (1–3, 5).

## 3. Minority stress, negative attitudes, discrimination, and mental health

Meyer's minority stress model identifies distal and proximal stressors as risk factors that can contribute to the higher rates of mental disorders among LGBTQ+ individuals (17). Internal processes, such as self-stigma and internalized homophobia, are considered proximal stressors (17). On the other hand, negative attitudes and discrimination, such as those experienced by LGBTQ+ Filipinos, are considered distal stressors or external objective stressful events (9–21). In the Philippines, evidence suggests that self-stigma, negative attitudes, and discrimination against LGBTQ+ Filipinos contribute to poor mental health, including their higher rates of suicidal ideations, depression, anxiety, and stress than the general population (1, 9–16, 18–21). Therefore, there is a need for measures that promote inclusive and affirming mental health for LGBTQ +. This is in keeping with the country's recent pledge to respond to the gendered needs of people with mental health concerns in its recently enacted Mental Health Act (22). Herewith, this opinion paper proposes several steps that can be undertaken to promote LGBTQ+ inclusive and affirming mental healthcare in the Philippines.

## 4. Discussion

### 4.1. Philippine professional organizations involved in mental healthcare can commit to LGBTQ+ inclusive and affirming mental healthcare practices

The past role of mental health professionals, particularly psychiatrists, in spreading the enduring stigma against LGBTQ+ people gives them a responsibility to affirm the diversity of SOGIE and oppose attempts to change it (24, 25). In recent years, the Philippine mental health professions also participated in this LGBTQ+ stigma promulgation (i.e., the promotion of Conversion therapy) (7, 10). Thus, there is a need for Philippine professional organizations involved in mental healthcare to uphold the rights and affirmation of LGBTQ+ identities and the diversity of SOGIE. By doing so, distal stressors that stem from mental health care can be avoided and reduced (1, 9–21). Furthermore, this assurance from professional organizations involved in mental healthcare is a welcome signal to LGBTQ+ Filipinos that their identities are respected in psychiatric and mental healthcare practices.

The Philippine Psychological Association (PAP) recently instituted its commitment to gender-affirming mental healthcare through its policy statement calling on psychologists “to ensure the advancement of LGBTQ+ rights and welfare” (10). Similarly, other Philippine professional organizations, such as the Philippine Psychiatric Association (PPA) and the Philippine Guidance and Counseling Association (PGCA), should also call on their members to uphold the commitment to advance LGBTQ+ inclusion, affirmation, and rights in their professional practice.

### 4.2. Professional organizations, higher education institutions, and hospitals in the Philippines can abolish the practice of conversion therapy and integrate LGBTQ-affirmative therapy in psychiatric and mental health training programs

Organizational commitment to LGBTQ+ inclusivity and affirmation in mental healthcare should be paralleled with actions among individual members. As a first step, Philippine professional organizations involved in mental healthcare need to abolish and oppose the practice of conversion therapy. Conversion therapy rejects an individual's inherent identity (24–27). Moreover, conversion therapy not only leads to poor mental health but may also violate a constitutionally guaranteed human right (24–27). Thus, psychiatrists and other mental health professionals must commit themselves to end this practice.

To further advance action at the individual level, psychiatrists and other mental health professionals should have the competency and understanding of gender affirmation and inclusivity in their practice (24, 25). However, in the Philippines, LGBTQ+ organizations have highlighted that few people are trained to address their mental health concerns (2). For instance, psychiatrists and other mental health professionals can train on and practice affirmative therapy, defined as “a type of psychotherapy used to validate and advocate for the needs of sexual and gender minority clients” (28). Likewise, promising sexual and gender-affirmative mental healthcare practices, such as affirmative psychotherapy principles and affirmative Cognitive Behavioral Therapy, can be studied, adapted, and expanded in the Philippines (29, 30). Affirmative Cognitive Behavioral Therapy, which validates stigmatized identities by acknowledging the impact of their sexual and gender identity-based stigma and targeting their cognitive, affective, and behavioral processes, has shown promise in combatting the increased rates of distress among LGBTQ+ individuals (29). Similarly, practicing affirmative psychotherapy principles, such as normalizing the impact of minority stress, decreasing avoidance, restructuring minority stress cognitions, and empowering and validating the unique strengths of LGBTQ+ individuals, may help them cope with minority stress, including internalized homophobia (30). These affirmative mental healthcare practices can be supplemented by studying and incorporating the effects of promising medical interventions, such as gender-affirmative hormone therapy (e.g., testosterone or estrogen), which have shown promising results in improving the psychosocial functioning of gender-diverse people (31). Thus, universities and colleges with mental health-related degree programs (i.e., counseling and psychology), as well as hospitals with psychiatric residency training programs, in the Philippines need to upscale and integrate gender-affirmative therapy into their training and research programs. Doing so can foster and nurture future generations of LGBTQ+ supportive psychiatrists and mental health professionals.

### 4.3. The Philippine government can include LGBTQ+ mental health as a national research agenda

While previous studies suggest that distal stressors contribute to poor mental health among LGBTQ+ Filipinos, they also highlighted that the determinants of mental health problems among LGBTQ+ Filipinos might vary from the general population (1, 8, 11–13, 18). This variation is accounted for by the unique cultural features of Philippine society, such as its colonial patriarchal norms (1–3, 8, 11–13). For example, a romantic relationship was found to be protective among heterosexual cisgender Filipinos but not among LGBTQ+ individuals (1). Thus, previous studies emphasized the need for further research to understand and address the mental health disparities among LGBTQ+ Filipinos (1, 11–16). This need is echoed by local LGBTQ+ organizations in a national dialogue with the UNDP and USAID, where they emphasized that there is poor information on their mental health (2). This limited information on LGBTQ+ mental health may reflect the apparent invisibility of LGBTQ+ Filipinos in the local mental health literature. Thus, moving their mental health agenda from the margins to the center is necessary to address their invisibility and mental health needs. As a start, the Philippine Department of Science and Technology, the government agency mandated to provide the direction and leadership of scientific and technological efforts in the country, can include LGBTQ+ research in the Philippines' National Research and Development Agenda under its mental health section.

### 4.4. Psychiatrists and other mental health professionals can advocate for LGBTQ+ rights and welfare

The Philippine society itself, with its heteronormative norms, negative attitudes, and continuous discrimination, can be a fertile ground for minority stress that accounts for the high rates of mental disorders among Filipinos with LGBTQ + (1–3, 5, 23). Hence, as patient advocates, psychiatrists and other mental health professionals in the Philippines need to advocate for a more gender-affirming society conducive to the mental health of all individuals, such as supporting the proposed SOGIE Equality Bill. By supporting this bill and the societal effort to decrease SOGIE-based discrimination and negative attitudes, psychiatrists and other mental health professionals take part in the systemic reduction of minority stress that may contribute to mental health disparities among LGBTQ+ Filipinos (1, 2, 8, 11–16, 18–21).

## 5. Conclusion

In general, Filipino psychiatrists and other mental health professionals should affirm and advocate the plurality of SOGIE at both the individual and the social levels. Among others, inclusive and affirming mental health and psychiatric services for LGBTQ + can be achieved in the Philippines by committing to gender inclusivity, opposing conversion therapy, integrating affirmative therapy for LGBTQ in training programs, including LGBTQ + mental health in the Philippine research agenda, and advocating for the rights and freedoms of these marginalized individuals in Philippine society. In doing so, the Philippines can be the country with the most gender equality in Asia, which is inclusive of the spectrum and diversity of sexualities and genders as well as conducive to mental health.

## Author contributions

RA had substantial contributions to the design, drafting, revision, acquisition, interpretation, and final approval of the data and work.
